# Pump quantum efficiency optimization of 3.5 μm Er-doped ZBLAN fiber laser for high-power operation

**DOI:** 10.1007/s12200-023-00089-w

**Published:** 2023-11-09

**Authors:** Lu Zhang, Shijie Fu, Quan Sheng, Xuewen Luo, Junxiang Zhang, Wei Shi, Jianquan Yao

**Affiliations:** 1 Institute of Laser and Optoelectronics, School of Precision Instrument and Optoelectronics Engineering, Tianjin University, Tianjin, 300072 China; 2 Key Laboratory of Opto-Electronic Information Technology, Ministry of Education, Tianjin University, Tianjin, 300072 China

**Keywords:** Fiber laser, Mid-infrared, Er-doped ZBLAN fiber, Dual-wavelength pumping

## Abstract

976 nm + 1976 nm dual-wavelength pumped Er-doped ZBLAN fiber lasers are generally accepted as the preferred solution for achieving 3.5 μm lasing. However, the 2 μm band excited state absorption from the upper lasing level (^4^F_9/2_ → ^4^F_7/2_) depletes the Er ions population inversion, reducing the pump quantum efficiency and limiting the power scaling. In this work, we demonstrate that the pump quantum efficiency can be effectively improved by using a long-wavelength pump with lower excited state absorption rate. A 3.5 μm Er-doped ZBLAN fiber laser was built and its performances at different pump wavelengths were experimentally investigated in detail. A maximum output power at 3.46 μm of ~ 7.2 W with slope efficiency (with respect to absorbed 1990 nm pump power) of 41.2% was obtained with an optimized pump wavelength of 1990 nm, and the pump quantum efficiency was increased to 0.957 compared with the 0.819 for the conventional 1976 nm pumping scheme. Further power scaling was only limited by the available 1990 nm pump power. A numerical simulation was implemented to evaluate the cross section of excited state absorption via a theoretical fitting of experimental results. The potential of further power scaling was also discussed, based on the developed model.

**Graphical Abstract:**

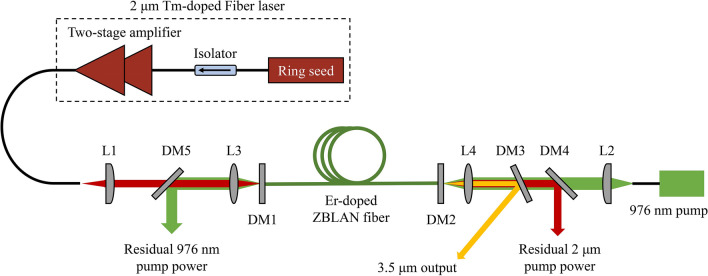

## Introduction

Mid-infrared lasers operating in the wavelength region of 3.5 μm have received a lot of attention due to their applications in defense, medical treatment and spectroscopic analysis [1]. In addition, this wavelength region is the so-called molecular fingerprint region consisting of many molecular absorption bands, which also makes 3.5 μm lasers attractive for gas sensing and polymer processing [2, 3].

3.5 μm lasing can be achieved in Er-doped fluoride fiber via the ^4^F_9/2_ → ^4^I_9/2_ transition. Early demonstrations of 3.5 μm Er-doped fluoride fiber lasers were mainly based on 0.65 μm single-wavelength pumping scheme, where the Er ions in ground state were directly excited to the 3.5 μm upper lasing level [4], followed by 3.5 μm transition. However, many Er ions accumulated in the lower long-lived levels (^4^I_11/2_ and ^4^I_13/2_) and could not return to the ground state, which reduced the pump absorption and resulted in an inefficient laser conversion. The maximum 3.5 μm laser output power obtained through such pumping scheme was only 10 mW [5]. In 2014, Henderson-Sapir et al. proposed a novel 0.98 μm + 1.97 μm dual-wavelength pumping scheme, aiming to address the bottlenecks resulting from ion accumulation in the ^4^I_11/2_ energy level [6]. In the dual-wavelength pumping scheme, the Er ions were first promoted to ^4^I_11/2_ level by the 0.98 μm ground state absorption (GSA). Taking this level as a virtual ground state (VGS), the second pump at 1.97 μm, which corresponded to the peak of ^4^I_11/2_ → ^4^F_9/2_ transition, was introduced to further promote the Er ions to ^4^F_9/2_ level via virtual ground state absorption (VGSA), creating a population inversion for the ^4^F_9/2_ → ^4^I_9/2_ transition and recycling the Er ions. It should be noted that the main role of the 0.98 μm pump was to provide the initial Er ions population in the VGS, and the 3.5 μm laser gain were almost entirely contributed by the 1.97 μm pump, thus offering benefit from a much lower quantum defect. Based on the dual-wavelength pumping scheme, Henderson-Sapir et al. demonstrated the first hundred-milliwatt-level 3.5 μm Er-doped ZBLAN fiber laser with a slope efficiency (with respect to 1.97 μm pump power) of ~ 25.4%. With the fabrication technique development of ZBLAN fibers and ZBLAN fiber-based devices, such as fiber Bragg gratings directly written in ZBLAN fiber, the 3.5 μm laser output power has been improved significantly in recent decade [7–10]. By optimizing the gain fiber lengths and output coupling ratio, a 15 W all-fiberized 3.5 μm Er-doped ZBLAN fiber laser was demonstrated by Lemieux-Tanguay et al. in 2021, which is up to now the highest output power in this wavelength region [11].

It should be noted that in a dual-wavelength pumping scheme, the absorption process of the 1.97 μm pump is accompanied with another excited state absorption (ESA) process centered at 1913 nm. Starting from the ^4^F_9/2_ level (the upper lasing level of the 3.5 μm laser), the ESA consumes the Er ion population and reduces the 3.5 μm laser gain. Such behavior reduces the pump quantum efficiency (the ratio of pump power consumed by the VGSA to the total absorbed 2 μm pump power) and even causes strong laser quenching, i.e., laser power decreases at insufficient 976 nm pumping. A high-power 976 nm pump is generally required to provide sufficient Er ion population in VGS, so that the absorption of the 1.97 μm pump is dominated by the ^4^I_11/2_ → ^4^F_9/2_ transition for a higher pump quantum efficiency. However, such an arrangement will inevitably decrease the overall optical efficiency (with respect to the total pump power of 0.98 and 1.97 μm). For example, in Ref. [11], only when the 976 nm pump power was higher than 50 W, was it possible for the 3.5 μm output power to maintain a linear increase without quenching, while the overall optical efficiency was only 17%.

Considering that the ESA process exhibits an absorption peak at ~ 1913 nm, longer pump wavelength results in decreased ESA cross section, which can reduce ESA-induced pump absorption and thus help to increase the pump quantum efficiency. On the other hand, even though the VGSA shows an absorption peak around 1976 nm, a broad absorption spectrum band extending to 2020 nm [12] offers a large tolerance for pump wavelength. Hence, there is a trade-off between strong pump absorption and high pump quantum efficiency. That is, optimizing the pump wavelength, to balance the VGSA and ESA processes, should be considered for high-efficiency and high-power 3.5 μm laser generation.

Here, we experimentally studied the effect of the VGSA pump wavelength on the behavior of a 3.5 μm fiber laser. A dual-wavelength pumped Er-doped ZBLAN fiber laser was demonstrated and three different VGSA pump wavelengths (1976, 1990, and 2004 nm) were investigated. As the 976 nm pump power was fixed at 5.2 W, a maximum 3.5 μm laser output of ~ 7.2 W was achieved with the 1990 nm pump laser, while further increase of the pump wavelength induced an output power degradation owing to the reduced pump absorption, despite the weaker ESA. A numerical model was developed thereafter to reproduce the experimental results and thus derive the ESA cross section at the above three wavelengths for further power scaling predication.

## Experimental setup

**Fig. 1:**
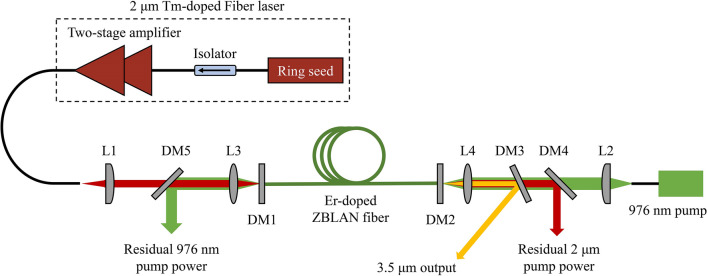
Experimental setup of the dual-wavelength pumped 3.5 μm Er-doped ZBLAN fiber laser. DM1 − DM5: dichroic mirrors; L1 and L2: aspheric lens; L3 and L4: CaF_2_ biconvex lens

Figure 1 shows the experimental setup of the 3.5 μm Er-doped ZBLAN fiber laser bidirectionally pumped at 976 nm and 2 μm. The 976 nm pump was a multimode laser diode (BWT Beijing Ltd.) with a maximum output power of 30 W. The 2 μm pump was generated from a homemade single-mode Tm-doped fiber laser based on MOPA configuration, with a ring-cavity oscillator seed and two stages of amplifiers. 3.7 m double-cladding Tm-doped fiber (Nufern, SM-TDF-10P/130-M) was used in the power amplifier, and cascaded with its matched passive fiber (Nufern, SM-GDF-10/130-15 M) for laser output. Both active and passive fiber allowed single-transverse-mode propagation above 1.96 μm, and this fact benefited a higher pump coupling efficiency. Three laser wavelengths (1976, 1990, and 2004 nm) could be obtained by changing the seed wavelength, and the maximum achievable laser powers at 1976, 1990, and 2004 nm were 28, 27.2, and 26.5 W, respectively. The 976 nm and 2 μm pump lasers were first collimated by two aspheric lens (L1: *f* = 15 mm, and L2: *f* = 8 mm) and then individually focused and coupled into the Er-doped ZBLAN fiber through two CaF_2_ lenses (L3 and L4: *f* = 12.7 mm), where the 976 nm pump and 2 μm pump laser were coupled into the inner cladding and the core of ZBLAN fiber, respectively. The gain fiber was a piece of 6.5 m Er-doped ZBLAN fiber (Le Verre Fluoré) with a core diameter of 16.5 μm and core numerical aperture of 0.125. It had a low doping concentration of 1 mol.%, which helped to reduce the thermal loads and ensure the laser stability. The cavity was constructed by butting the Er-doped ZBLAN fiber to two dichroic mirrors (DM1 and DM2), both of which were high-transmissive (*T* ~ 96%) at 976 nm and 2 μm. The reflectivity of DM1 and DM2 at 3.5 μm were ~ 98% and ~ 25%, respectively. Thus, the DM2 serves as output coupler to extract the intracavity laser energy. Both fiber end facets were perpendicularly cleaved by a large diameter fiber cleaver (Vytran, LDC-400); this ensured a low-loss butted joint and high coupling efficiency. Another dichroic mirror (DM3) with the same coating as the DM1, was placed after L2 to split the 3.5 μm laser from the pump laser. The plane of the DM3 is at around 20° from perpendicular to the system axis. The residual 2 μm pump power was reflected out of the optical path by a 45° dichroic mirror (DM4), which had a high reflectivity (> 95%) around 2 μm and a high transmission at 976 nm (> 95%). Another 45° dichroic mirror (DM5), which had a high reflectivity (> 95%) at 976 nm and a high transmission around 2 μm (> 95%) was inserted between L1 and L3 to reflect out the residual 976 nm pump power. No active cooling management was employed in the experiment and all of the Er-doped ZBLAN fibers are coiled into a ~ 30 cm ring and laid on an aluminum plate for passive heat dissipation.

Unlike in high-power 2.8–3 μm fiber lasers, no endcaps were needed in this 3.5 μm fiber laser since it does not operate in the OH absorption band. Moreover, the current laser was constructed based on a spatial configuration; both of pump incidence and laser feedback relied on the light coupling at fiber facet. The introduction of endcap would inevitably reduce the pump coupling efficiency and cavity feedback, and would also decrease the laser efficiency and hinder the power scaling.

## Experimental results

**Fig. 2:**
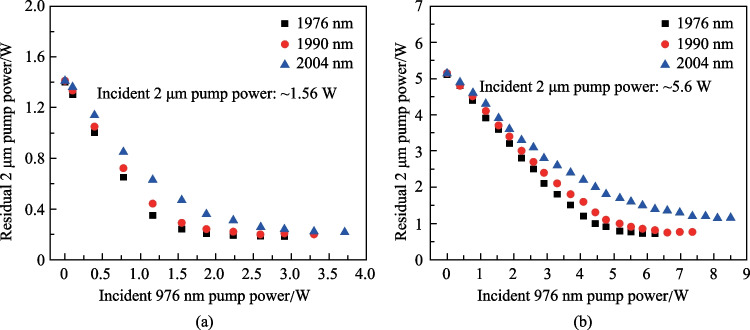
Measured residual 2 μm pump power as a function of incident 976 nm pump power at incident 2 μm pump power of **a** 1.56 W, and **b** 5.6 W

Under an open cavity (removing DM1 and DM2) without lasing, we first measured the absorption of 976 nm cladding pump power and 2 μm core pump power. At the 976 nm pump powers of 2.7, 3.5, and 5.2 W, the residual 976 nm pump powers were 1.13, 1.44, and 2.2 W, respectively, corresponding to a cladding absorption of around 58%. It is also found that the 2 μm pump had little effect on the 976 nm pump absorption. Figure 2 shows the residual 2 μm pump power at the three specific wavelengths as a function of 976 nm pump power. The incident pump powers at 2 μm were set to be 1.56 and 5.6 W in Fig. 2a and b, respectively. Note that the perpendicularly cleaved fiber end facet could provide a wavelength-independent reflectivity of ~ 4%, indicating that only ~ 96% of 2 μm pump power was actually coupled into the fiber. It was found that without the 976 nm pump, ~ 91% of 2 μm pump power could transmit through the Er-doped ZBLAN fiber. In this case, the 2 μm pump power cannot be absorbed due to the very limited accumulation of Er ions in the VGS, the power drop was mainly attributed to the background loss of the fiber. Increasing the 976 nm pump power would effectively populate the ^4^I_11/2_ level, leading to a strong absorption of 2 μm pump power. As shown in Fig. 2a, a long-wavelength pump exhibited a higher residual pump power due to the diminishing VGSA cross section, and this effect was further amplified by increasing the incident 2 μm pump power, as shown in Fig. 2b. At an incident 2 μm pump power of ~ 5.6 W, the minimum residual pump powers at three wavelengths were 0.72, 0.77, and 1.1 W, corresponding to fractional absorption of 86%, 85%, and 78.5%, respectively. Note that when the 976 nm pump power was high enough, the pump absorption at 2004 nm did not decrease much in spite of a 30 nm deviation from the peak of VGSA. The residual 2 μm pump power at high 976 nm pump power can be attributed to the facts that: (1) the Er-doped ZBLAN fiber used here allowed multimode propagation at ~ 2 μm; (2) the focused beam diameter was larger than the core diameter and part of 2 μm pump laser was coupled into the inner cladding. Based on the above results, the effective fiber core coupling efficiency (with respect to the 2 μm pump power launched from Tm-doped fiber laser) was estimated to be around 82%. The coupling efficiency of the 976 nm cladding pump was also measured using a passive matched ZBLAN fiber (Le Verre Fluoré). The fiber had an inner cladding diameter of 250 μm and cladding numerical aperture of 0.45, and these values were very close to those of the Er-doped ZBLAN fiber. The coupling efficiency was measured to be around 94%.

The laser spectrum was measured by an optical spectrum analyzer (OSA, Bristol, 771B-MIR). The cavity mirrors (DM1 and DM2) provided a broad reflection band (3.3–4 μm), so a strong wavelength drift was observed in experiment. That is, the operating wavelengths during two measurements at same pump power might be completely different. We recorded the laser wavelength over 50 measurements at different pump powers and pump wavelengths. It was found that the laser would dominantly operate within the wavelength region of 3.45–3.47 and 3.52–3.55 μm, but sometimes the lasing wavelength randomly shifts above 3.6 μm. The longest laser wavelength observed in experiment was ~ 3.66 μm. Such wavelength drift can be attributed to the variation of Er ions distribution in sublevels [13]. It was also found that as the wavelength rose above 3.6 μm the output power dropped sharply due to a reduced emission cross section.

**Fig. 3.**
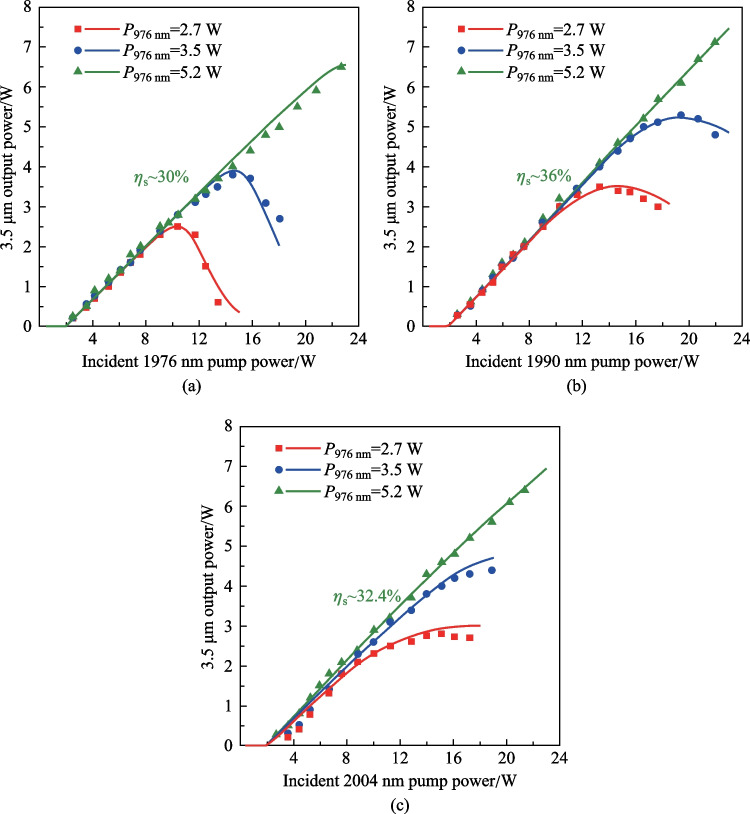
3.5 μm output power as a function of incident 2 μm pump power under different 976 nm pump power as the Er-doped ZBLAN fiber laser was pump at **a** 1976 nm, **b** 1990 nm, and **c** 2004 nm. The scatters and curves represent the experimentally measured results and calculated results based on the numerical model in Sect. 4

To investigate the effect of 2 μm pump wavelength on the 3.5 μm power behavior, we measured the 3.5 μm output power and residual 2 μm pump power when the laser was pumped at 1976, 1990, and 2004 nm. Considering the potential influence of wavelength fluctuation on output power, the measurement was repeated several times and the data were recorded only when the Er-doped ZBLAN fiber laser was operated at ~ 3.46 μm, i.e., at the emission peak of Er ions. The long-term stability of output power was also measured at different pump power and each measurement lasted 5 min. For all the cases, the root mean square fluctuation of output power was below 2.1%, demonstrating stable operation. The output power as a function of incident 1976 nm pump power under different 976 nm pump power is shown in Fig. 3a. Strong laser quenching was observed at lower 976 nm pump power (2.7 and 3.5 W). As discussed above, such behavior originated from the ESA and can be suppressed by increasing the Er ions population in ^4^I_11/2_ level, i.e., by increasing the incident 976 nm pump power. A linear power increase with a maximum output power of 6.5 W was achieved as the 976 nm pump power was increased to 5.2 W. Figure 3b and c show the 3.5 μm output power evolution of Er-doped ZBLAN fiber laser pumped at 1990 and 2004 nm, respectively. Note that at these two pumping wavelengths, the rollover of output power under higher 2 μm pump was delayed and the decrease of 3.5 μm output power also became much slower. In particular for the 2004 nm pumping condition, the 3.5 μm output power tended to saturate rather than decrease. Such behavior indicates that the laser quenching was mitigated to some extent, which can be attributed to the fact that long wavelength pump decreased the ESA-induced loss, and enhanced the gain of 3.5 μm lasing. With the pump laser at 1990 nm, the maximum 3.5 μm output power was improved to 7.2 W at 976 nm pump power of 5.2 W, corresponding to a slope efficiency (with respect to the incident 1990 nm pump power) and overall optical efficiency of ~ 36% and 26.2%, respectively. However, at the same 976 nm pump power, the maximum 3.5 μm output power achieved with 2004 nm pump scheme decreased to 6.3 W, though pump at 2004 nm benefited a much weaker ESA given that the absorption cross section of ^4^F_9/2_ → ^4^F_7/2_ transition decreased monotonically in the long-wavelength region.

**Fig. 4:**
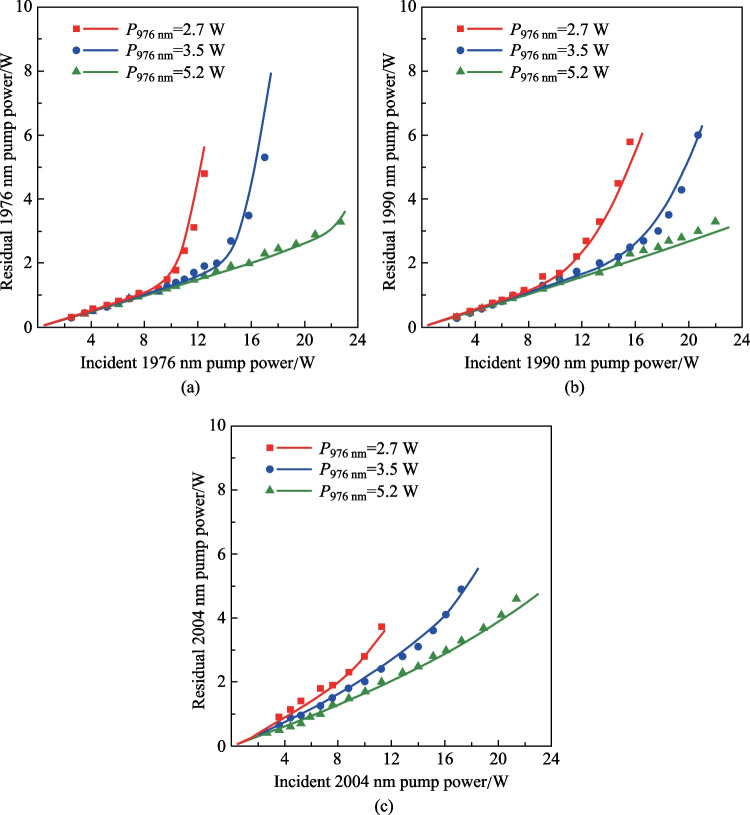
Residual 2 μm pump power as a function of incident pump power at the specific wavelengths of **a** 1976, **b** 1990, and **c** 2004 nm under different 976 nm pump power. The scatters and curves represent the experimentally measured results and calculated results based on the numerical model in Sect. 4

The decreased 3.5 μm output power at 2004 nm pump can be understood in terms of the evolution of residual 2 μm pump power as shown in Fig. 4. Deficiency in 976 nm pumping led to a surge of residual 2 μm pump power, which was ascribed to the depletion of Er ions in VGS. Note that the increase of residual 2 μm pump power was even faster than that of the incident pump power. Such behavior can be understood as follows: As the Er ions in VGS were depleted, further increasing 2 μm pump power would only enhance the ESA process, making more Er ions in the ^4^F_9/2_ level be excited to higher levels. These Er ions could not return to the VGS via the fast 3.5 μm emission and non-radiative decay. In other words, the available Er ions in VGS were reduced at higher 2 μm pump power, which decreased the 2 μm pump absorption and resulted in a much faster increase of the residual 2 μm pump power. By increasing 976 nm pump power to provide sufficient initial Er ions population, the surge could be eliminated and the residual 2 μm pump power can maintain linear increase. In 1976 and 1990 nm pumping cases, the maximum residual pump power was around 3.3 W, agreeing well with the absorption measurements in Fig. 2. Note that the 976 nm pump power required to deplete the 2 μm pump power was much lower than that without cavity feedback. This can be attributed to the fact that the 3.5 μm lasing here also supplied the VGS population and moderated the requirement of GSA. At a longer pump wavelength with weak ESA, the surge became less significant and the residual pump power increased with a higher slope due to the reduced absorption cross section of VGSA. At 976 nm pump power of 5.2 W, a maximum 2004 nm pump power of 4.6 W was residual, which was much higher than those in 1976 and 1990 nm pumping schemes and resulted in the decrease of 3.5 μm output power. A higher slope efficiency and output power could be anticipated by increasing the Er-doped ZBLAN fiber length for sufficient pump absorption.

**Fig. 5:**
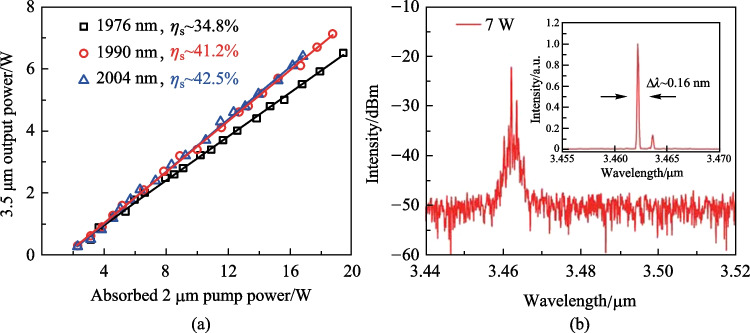
**a** 3.5 μm output power as a function of absorbed 2 μm pump power at 976 nm pump power of 5.2 W; **b** laser spectra at 7 W output power; inset: zoomed spectrum in linear scale. The scatters and curves in **a** represent the experimentally measured results and the corresponding linear fitting

Based on the residual 2 μm pump power, the 3.5 μm laser power as a function of the absorbed 2 μm pump power was calculated for the 5.2 W, 976 nm pump and the results are shown in Fig. 5a. The highest slope efficiency of ~ 42.5% was achieved at 2004 nm pumping, while the slope efficiencies under 1990 and 1976 nm pump decreased to 41.2% and 34.8%, respectively. This indicates that using a long-wavelength 2 μm pump, although deviating from the peak position of VGSA, could effectively alleviate the ESA-induced loss and make full use of pump. In other words, more Er ions promoted to ^4^F_9/2_ level would priorly participate in the 3.5 μm emission rather than being excited to other higher energy levels. However, such efficiency improvement was offset and even overweighed by insufficient pump absorption due to the reduced VGSA cross section, making the output power start to decrease when an over-long wavelength was used. Moreover, a small VGSA cross section indicated that more 976 nm and 2 μm pump power were required to achieve sufficient pump absorption, which resulted in a higher heat load at the pump incident port. Based on the above discussion, we believe that 1990 nm could be a preferred wavelength for efficient 3.5 μm laser generation since sufficient pump absorption and weak quenching were both realized by balancing the VGSA and ESA. Figure 5b shows the laser spectrum recorded at 7 W output power. The center wavelength was 3462 nm with an optical signal to noise ratio (OSNR) of ~ 28 dB. The inset shows the zoomed spectrum with linear scale, which demonstrates an linewidth of around 0.16 nm (full width at half maximum).

We used a thermal camera (Agilent, U5855A) to measure the Er-doped ZBLAN fiber temperature. At the maximum pump power (5.2 W @ 976 nm, 23 W @ 1990 nm), it was found that the first ~ 5 cm fiber from the 976 nm pump incident port, which experienced the strongest 2 μm pump absorption, exhibited the highest temperature of ~ 42 ℃. In this case, no fiber damage was observed, revealing that the passive heat dissipation in the current design was already enough to achieve stable laser operation. For further power scaling to 10 W class or higher, efficient thermal managements, like actively air cooling and water cooling, should be added to mitigate the heat issue. Furthermore, given that fluoride fiber-based combiners will become available in future, dual-end pumping scheme is also a promising approach for spreading the heat load.

## Numerical modeling and calculation

### Rate equation model

**Fig. 6:**
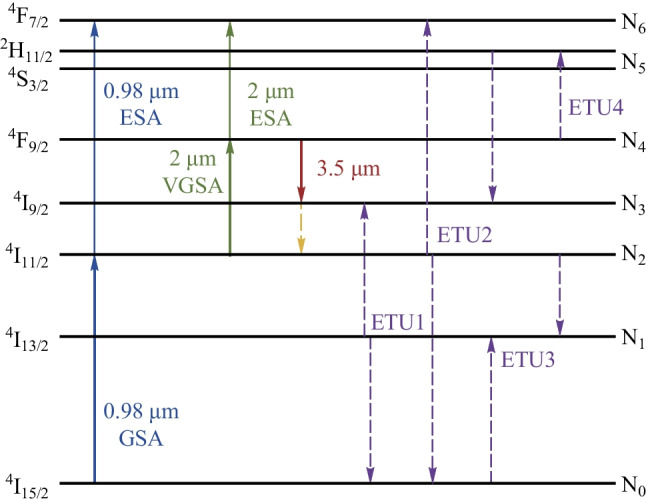
Energy level diagram and some important transitions of 976 nm + 2 μm dual-wavelength pumped 3.5 μm Er-doped ZBLAN fiber laser. GSA: ground state absorption; VGSA: virtual ground state absorption; ESA: excited state absorption; ETU: energy transfer up-conversion

Figure 6 shows the energy level diagram of Er ions in the ZBLAN host and some transitions involved in the 3.5 μm lasing. Based on the rate equations and power propagation equations in Ref. [14], a numerical model was developed in this work. Unlike in the previous theoretical works, we here redefine the power filling factor of 2 μm pump. Based on the knife-edge method, the diameter of the focused beam spot of the 2 μm pump after L3 was measured as ~ 18 μm, which was a bit larger than the fiber core size, indicating that a fraction of 2 μm pump laser was inevitably couple into the inner cladding. The pump absorption was dominated by the pump laser coupled into the fiber core; for pump laser coupled into the inner cladding, the absorption coefficient and contribution to the laser gain were much smaller than those of pump laser coupled into the core. In other words, the pump laser inside the inner cladding and the pump laser trapped in fiber core underwent very different absorption processes, so that the effective power filling factor could not be characterized with one single value. Here, we divided the incident 2 μm pump power into two parts, i.e., *P*_core_ and *P*_cladding_, for the fiber core and inner cladding, respectively. Based on the absorption measurement (Fig. 2) and spot size, *P*_core_ and *P*_cladding_ were assumed to account ~ 85% and ~ 15% of the total incident pump power, respectively. For *P*_core_, its power filling factor can be calculated by [15]:

1 $${\Gamma } = 1 - \exp \left[ - 2(\frac{{r_{{{\text{core}}}} }}{\omega })^{2} \right],$$

where *r*_core_ and *ω* are the radius of the fiber core and mode field, respectively. The latter can be calculated based on the Marcuse empirical formula where the core mode field is presumed to have a Gaussian profile [16].

For *P*_cladding_, the power filling factor, which is same as that of 976 nm pump, was calculated as the ratio of the core area to that of the inner cladding. Note that the fiber used in our experiment had a double D-shaped inner cladding, and such asymmetrical design can increase the cladding pump absorption, resulting the effective inner cladding diameter being smaller than its geometrical size. Maes et al. carried out a theoretical investigation to fit the experimental results of a 3.5 μm laser system with the same Er-doped ZBLAN fiber [14], where the best fit was realized with an effective inner cladding diameter of ~ 170 μm. Here we used this value to calculate the power filling factor of *P*_cladding_ and 976 nm pump power.

The other modification was the boundary condition of the signal. Considering the thermal expansion of ZBLAN glass under high laser power, in our laser arrangement, the fiber end facets were not closely butted to the DM but remained at a distance of around 15 μm to avoid potential thermal damage. Any non-collimated arrangement, e.g., a tilted mirror, would introduce significant cavity loss and result in output power degradation. Here, a scale factor *k*, which is defined as the ratio of the signal power coupled back into the fiber core to the signal power launched from the fiber, was introduced to characterize such feedback loss. For simplicity, the feedback loss at HR (High reflectivity) port and OC (Output coupling) port were assumed to be identical and were characterized by one same scale factor. The modified boundary conditions of signal are given by

2 $$P_{{\text{s}}}^{ + } (0) = P_{{\text{s}}}^{ - } (0)R_{1} k,$$

3 $$P_{{\text{s}}}^{ - } (L) = P_{{\text{s}}}^{ + } (L)R_{2} k,$$

where *P*_s_ denotes the intracavity signal power and the superscript “+” and “−” denote the forward and backward propagating direction, respectively. *R*_1_ and *R*_2_ are the reflectivity of the HR DM and OC DM at the signal wavelength, respectively.

Except for the above two modifications, all other parameters, like lifetime, branching ratios and cross section, were identical to those used in Ref. [14]. Here, we used a coupled solution method reported in Ref. [17] to solve this two-point boundary value problem. The whole Er-doped ZBLAN fiber was spatially discretized into equally spaced segments each with length of 1 cm. At each segment, the rate equations group was solved by the stiff ode15s solver to obtain the population densities in each level. Afterwards, based on the population densities distribution, the power evolution along the fiber could be obtained by solving power evolution equation group with the non-stiff ode45 solver.

### ESA cross section

It should be noted that the spectrum shape of the ESA process, especially the specific values of the cross section, are still unknown and are difficult to measure directly. The characterization of such ESA processes is generally based on a theoretical tool as presented in Ref. [14], where a rate equation model was developed by Maes et al. and the cross section at 1976 nm was deducted by constructing a numerical fitting of the experimental data points. Here, the same method was used to calculate the ESA cross section at the three investigated pump wavelengths. The best fitting of the simulation result with the experimental data was achieved with a scale factor of 0.9, which indicates that a signal coupling loss of ~ 0.5 dB should be included. As shown in Figs. 3 and 4, the numerical model could well reproduce the 3.5 μm output power evolution and residual 2 μm pump power with ESA cross sections of ~ 0.8 × 10^–26^, ~ 0.4 × 10^–26^, and ~ 0.2 × 10^–26^ m^2^ at 1976, 1990, and 2004 nm, respectively. It could be found that the ESA cross section at 1976 nm obtained here is very close to the value (0.7 × 10^–26^ m^2^) reported in Ref. [14]. Considering the uncertainty introduced by different laser arrangements, we believe such a minor difference is fully acceptable and the values obtained here can be used for further laser performance analysis.

### Pump quantum efficiency

It was necessary to investigate the pump quantum efficiency of the current laser to clarify the improvement achieved via pump wavelength optimization. However, the exact absorbed pump power by VGSA was difficult to measure directly since the VGSA and ESA occurred almost simultaneously. Here, we adopted a theoretical tool to calculate this value. For calculation, the ESA was switched off and the pump absorption was completely contributed by the VGSA. In this case, the calculated slope efficiency (with respect to the absorbed pump power) can be regarded as the effective conversion efficiency from VGSA pump power to 3.5 μm output power for the current laser condition, i.e., establishing a quantitative relation between VGSA pump power and 3.5 μm output power. Based on the measured slope efficiency *η*_1_ and calculated slope efficiency *η*_2_, the pump quantum efficiency *η*_PQE_ can be obtained as follows:

4 $$\eta_{1} = \frac{{\Delta P_{{{\text{output}}}} }}{{\Delta P_{{\text{Total-abs}}} }},$$

5 $$\eta_{2} = \frac{{{\Delta }P_{{{\text{output}}}} }}{{{\Delta }P_{{\text{VGSA-abs}}} }},$$

6 $$\eta_{{{\text{PQE}}}} = \frac{{{\Delta }P_{{\text{VGSA-abs}}} }}{{{\Delta }P_{{\text{Total-abs}}} }} = \frac{{\eta_{1} }}{{\eta_{2} }},$$

where *P*_output_ is the 3.5 μm output power, *P*_Total-abs_ is the total absorbed pump power measured in the experiment, *P*_VGSA-abs_ is the calculated pump power absorbed via VGSA.

**Fig. 7:**
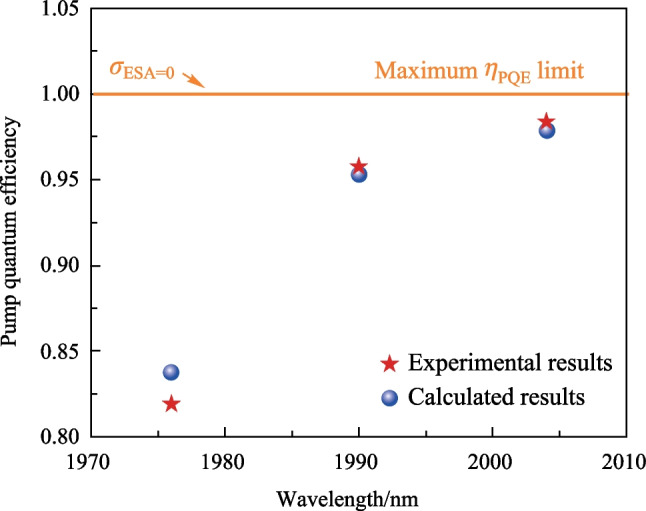
Calculated and experimental results of pump quantum efficiency at different pump wavelengths

Figure 7 shows the calculated pump quantum efficiency at different pump wavelengths for the 976 nm pump power of 5.2 W. In the ideal case where ESA is switched off, the pump absorption is entirely contributed by VGSA, leading to a maximum pump quantum efficiency of 1. This value can be considered as the pump quantum efficiency limit for dual-wavelength pumped 3.5 μm Er-doped fiber lasers with similar design. As expected above, a long wavelength exhibited a higher pump quantum efficiency, which increased from 0.819 (1976 nm) to 0.957 (1990 nm) and 0.984 (2004 nm). The pump quantum efficiency obtained with the calculated *η*_1_ were also present (blue solid circle) and the comparison with experimental results demonstrates a good agreement, as shown in Fig. 7. Note that the pump quantum efficiency achieved in the 2004 nm pumping case had almost approached the limit value, indicating that the effect of ESA was almost marginal at this wavelength, which could also be evidenced by the power behavior in Fig. 3c. A higher pump quantum efficiency can be expected with a longer pump wavelength; however, the decreased pump absorption resulting from the diminishing VGSA cross section should also been taken into account for laser efficiency optimization. For addressing such dilemma, one strategy is to find a new glass host to modify energy structure of Er ion to achieve a complete separation between the VGSA band and the ESA band.

### Potential further power scaling

**Fig. 8:**
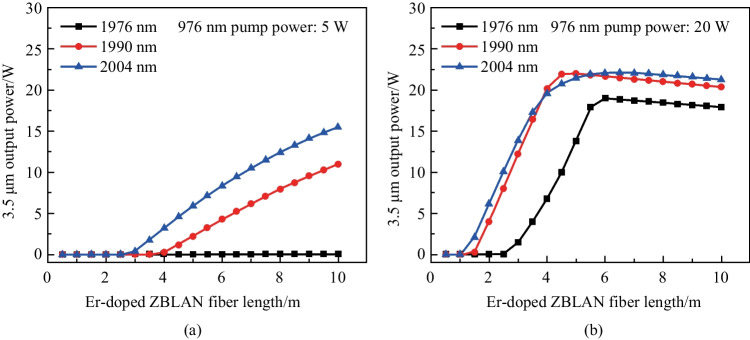
Calculated 3.5 μm output power as a function of Er-doped ZBLAN fiber length at 976 nm pump power of **a** 5 W, and **b** 20 W

Except for the 2 μm ESA process, the 3.5 μm laser performance could also be affected by many other parameters, such as 976 nm pump power, Er-doped fiber length, and output coupling ratio. This indicated that the optimal 2 μm pump wavelength may vary under different experimental conditions. In order to provide guidance for future power scaling of the 3.5 μm Er-doped ZBLAN fiber laser, we calculated the laser output power as a function of Er-doped ZBLAN fiber length for the above three pump wavelength cases with a fixed pump power of 50 W. Here, the modeling was implemented under an ideal all-fiber configuration, where all the coupling loss were neglected. Figure 8 shows the calculated results under 976 nm pump power of 5 and 20 W. As shown in Fig. 8a, at lower 976 nm pump power, pumping at 1976 nm was unable to achieve 3.5 μm lasing with any fiber lengths. This was because the 976 nm pump power was too low to realize sufficient pump absorption, and high 1976 nm pump power depleted the Er ions in ^4^F_9/2_ level by a strong ESA process. For 1990 and 2004 nm pumping cases, stable laser operation could be achieved at Er-doped ZBLAN fiber above ~ 3 and ~ 4 m, respectively. As shown in Fig. 8b, increasing 976 nm pump power significantly reduced the Er-doped fiber lengths needed for 3.5 μm lasing, which were ~ 1, ~ 1.5, and ~ 2.5 m for the 1976, 1990, and 2004 nm pumping cases, respectively, at 976 nm pump power of 20 W. These calculated results reveal that in the case with short Er-doped ZBLAN fiber or low 976 nm pump power, a long-wavelength pump is preferred. In other words, the employment of a long-wavelength pump allows us to use short Er-doped ZBLAN fiber and low-power 976 nm pump to achieve efficient 3.5 μm lasing, which is cost-effective and benefits higher optical efficiency. The maximum output powers were achieved at the correct fiber length which allowed the 2 μm pump power to be depleted completely. After this position, the output powers started to drop owing to the background loss of the ZBLAN fiber. This behavior also highlights the advantage of using short fiber and long-wavelength 2 μm pump.

## Conclusion

We study the effect of VGSA wavelength on the performance of the dual-wavelength-pumped Er-doped ZBLAN fiber laser at 3.5 μm. It is demonstrated that using a long-wavelength VGSA pump, which has a small ESA cross section, can help to suppress the laser quenching and improve laser efficiency. Nevertheless, there is a trade-off because an over-long wavelength may limit the pump absorption and resultant output power. By shifting the pump wavelength from 1976 to 1990 nm, the laser yields a maximum output power of 7.2 W with a slope efficiency of 41.2% (with respect to the absorbed 1990 nm pump power), 7.2 W highest output power and 41.2% efficiency are the highest achieved in such spatial-structured 3.5 μm Er-doped fluoride fiber lasers, to the best of our knowledge. For further power scaling at higher pumping conditions, our modeling shows that a high laser efficiency can be well maintained based on this pump wavelength and the optimization of cavity design. Furthermore, a numerical modeling is developed to reproduce the experimental results and characterize the ESA at three investigated wavelengths. Based on the obtained cross section, the further power scaling is discussed and the calculated results demonstrate the improvement of 3.5 μm output power via wavelength optimization.

## Data Availability

The data that support the findings of this study are available from the corresponding author, upon reasonable request.
